# Evaluation of the Impact of Pregnancy-Associated Factors on the Quality of Wharton’s Jelly-Derived Stem Cells Using SOX2 Gene Expression as a Marker

**DOI:** 10.3390/ijms23147630

**Published:** 2022-07-10

**Authors:** Paulina Gil-Kulik, Małgorzata Świstowska, Arkadiusz Krzyżanowski, Alicja Petniak, Anna Kwaśniewska, Bartosz J. Płachno, Dariusz Galkowski, Anna Bogucka-Kocka, Janusz Kocki

**Affiliations:** 1 Department of Clinical Genetics, Medical University of Lublin, 11 Radziwillowska Str., 20-080 Lublin, Poland; malgorzata.swistowska@umlub.pl (M.Ś.); alicja.petniak@umlub.pl (A.P.); janusz.kocki@umlub.pl (J.K.); 2 Department of Obstetrics and Pathology of Pregnancy, Medical University of Lublin, 11 Staszica Str., 20-081 Lublin, Poland; arkadiusz.krzyzanowski@umlub.pl (A.K.); anna.kwasniewska@umlub.pl (A.K.); 3 Department of Plant Cytology and Embryology, Institute of Botany, Faculty of Biology, Jagiellonian University in Kraków, 9 Gronostajowa St., 30-387 Cracow, Poland; bartosz.plachno@uj.edu.pl; 4 Pathology and Laboratory Medicine, Rutgers Robert Wood Johnson Medical School, New Brunswick, NJ 08901, USA; galkowd@fastmail.fm; 5 Chair and Department of Biology and Genetics, Medical University of Lublin, 4a Chodźki St., 20–093 Lublin, Poland; anna.kocka@umlub.pl

**Keywords:** SOX2, mesenchymal stem cells, gene expression, WJSC

## Abstract

SOX2 is a recognized pluripotent transcription factor involved in stem cell homeostasis, self-renewal and reprogramming. It belongs to, one of the SRY-related HMG-box (SOX) family of transcription factors, taking part in the regulation of embryonic development and determination of cell fate. Among other functions, SOX2 promotes proliferation, survival, invasion, metastasis, cancer stemness, and drug resistance. SOX2 interacts with other transcription factors in multiple signaling pathways to control growth and survival. The aim of the study was to determine the effect of a parturient’s age, umbilical cord blood pH and length of pregnancy on the quality of stem cells derived from Wharton’s jelly (WJSC) by looking at birth weight and using *SOX2* gene expression as a marker. Using qPCR the authors, evaluated the expression of *SOX2* in WJSC acquired from the umbilical cords of 30 women right after the delivery. The results showed a significant correlation between the birth weight and the expression of *SOX2* in WJSC in relation to maternal age, umbilical cord blood pH, and the length of pregnancy. The authors observed that the younger the woman and the lower the umbilical cord blood pH, the earlier the delivery occurs, the lower the birth weight and the higher *SOX2* gene expression in WJSC. In research studies and clinical applications of regenerative medicine utilizing mesenchymal stem cells derived from Wharton’s Jelly of the umbilical cord, assessment of maternal and embryonic factors influencing the quality of cells is critical.

## 1. Introduction

Mesenchymal stem cells (MSCs), especially obtained from perinatal tissues, including Wharton’s jelly of umbilical cord have been an excellent source of stem cells used in regenerative medicine during the last decade. MSCs derived from Wharton’s jelly (WJSC) possess a number of valuable properties including the high ability of self-renewal and differentiation, fast proliferation rate, immunosuppressive and paracrine properties and high expression of factors specific to embryonic stem cells e.g., SOX2 or POU5F1. Additionally, the umbilical cord is considered to be a safe and abundant resource for MSCs, and sampling does not raise any ethical concerns. In recent years WJSC have been the subject of numerous studies in both pre-clinical and clinical phases [1,2]. However, there is still a lack of in-depth information regarding the mechanisms affecting the regulatory properties of these cells, as well as the factors determining their therapeutic potential. The study focused on the evaluation of one expression of the pluripotency factors—SOX2 expression, at the mRNA level. The SOX2 gene (*SRY-Related HMG-Box Gene 2*) belonging to the SOX gene family was discovered and described by Stevanovic et al. in 1994. It has been mapped to 3q26.3-27 on the long arm of chromosome 3 [3]. It encodes the SOX2 protein consisting of 317 amino acids which contain the HMG domain, characteristic of all SOX proteins. The domain binds to the ATTGTT motif in the DNA [4,5,6,7]. In embryonic stem cells, the level of expression of transcription factors SOX2, OCT4 and NANOG impacts self-renewal, maintenance of pluripotency and reprogramming of somatic cells [8,9,10]. It also directs the differentiation of pluripotent stem cells to neural progenitors, as well as maintaining the properties of neural progenitor cells [11]. SOX2 is a vital gene participating in maintaining cell pluripotency [9,11]. Its mechanism of action involves activation and maintenance of the OCT4 gene expression [12]. The SOX2 protein, when interacting with the OCT4 forms a heterodimeric complex responsible for activation and silencing transcription processes of genes in control of cell differentiation [13]. The cell remains undifferentiated if there is an adequate level of SOX2 and even small changes in the SOX2 and OCT4 gene expression may cause the loss of pluripotency [11]. A drop in SOX2 expression leads to the differentiation of cells into trophectoderm [14], while its increase causes differentiation into meso-, ecto- and trophectoderm [15]. There are numerous reports recording the presence of SOX2 gene expression in human stem cells derived from different tissues, including perinatal [16,17,18], adipose [19,20], bone marrow [19,21], dental pulp [22], mammary gland [23,24] skin and heart muscle [20]. Many factors may influence gene expression and SOX2 protein level. The effect depends, among other factors, on the type of cell and the degree of differentiation. SOX2 protein concentration is regulated at many levels, including transcription, post-transcription and post-translation [25]. Horizontal gene transfer occurs through a process of self-regulation involving feedback mechanism, as well as mutual cross-regulation between SOX2 and OCT4, OCT4 and NANOG [26]. Current research indicates that the high proliferation potential is caused (at least partially) by the inhibitory effect of *SOX2* on genes involved in cell cycle regulation such as *CCND1* and *CDK4* genes [27]. 

SOX2 is considered irreplaceable and is required for the normal functioning of stem cells and their maintenance in an undifferentiated state as well as self-renewal [28]. In addition, it has been shown that SOX2 also affects migration and cell adhesion [29], SOX2 pituitary stem cells can hold additional roles in tissue expansion and homeostasis, acting as paracrine signaling centers to coordinate the proliferation of neighboring cells [30]. Research suggests that the presence of high levels of SOX2 is related to the therapeutic potential of stem cells, and the prospect of modulating SOX2 expression to achieve therapeutic benefit seems to be promising [31]. Understanding the molecular mechanisms governing *SOX2* functions will facilitate the use of pluripotent stem cells for clinical and biomedical applications, with particular emphasis on the modeling and treatment of various neurological disorders [11].

Factors influencing the *SOX2* gene expression in stem cells are still being intensively studied and there is a high demand for more data. The discovery of new factors influencing the expression of *SOX2* may contribute to better utilization of the cells taken from the patient, as well as better preparation and maintenance of cells in in vitro culture. 

The effect of maternal and neonatal factors on the quality of umbilical cord blood stems cells have been a subject of numerous studies [31,32,33,34,35,36,37,38,39,40]. One of the deciding factors in the usefulness of cord blood is the number of CD34+ stem cells. Studies involving umbilical cord blood stem cells have shown, inter alia, that the number of CD34+ cells depends on the number of previous live births and the body weight of the newborn, and is better with good weight and first babies, and decreases with subsequent births [41]. It was also observed in several studies that the gestational age negatively correlates with the number of CD34+ cells [42,43], and the number of CD34+ stem cells is significantly higher in premature babies [44]. Bielec–Berek et al. assessed the correlations between maternal age and selected properties of umbilical cord blood stem cells. Moreover, in this study, the correlation between the mode of delivery, the age of the mother and the quality of the obtained material for transplantation was assessed. The older the women from whom umbilical cord blood was collected, the lower the mean concentration of HSC cells in the material [37]. Aufderhaar et al. showed that perinatal factors such as low blood pH and prolonged first stage of labor correlate with increases in CD34+ cells and cord blood progenitor cells [45]. There is insufficient research related to the influence of these factors on the quality of mesenchymal stem cells of Wharton’s jelly of the umbilical cord. Available literature does not provide sufficient information on the influence of maternal age, birth weight and duration of pregnancy or the physicochemical properties of cord blood on the expression of the *SOX2* gene in stem cells derived from umbilical cord Wharton’s jelly. Therefore, in our work, we have decided to evaluate these factors. The study aims to bring closer the knowledge of MSC biology, the combination of in vivo factors necessary to maintain the state of undifferentiation, self-renewal potential and stem cell proliferation. Finding the optimal maternal age and other perinatal parameters for collecting and banking the highest quality material.

In view of the potential therapeutic benefits that may result from mesenchymal stem cell transplantation widening viewers, understanding how to maintain cell viability and in vitro pluripotency trait is of primary importance, bridging the gap in the knowledge of the specific origin of MSCs, from Wharton’s jelly as opposed to into umbilical cord blood.

The aim of the study was to assess the expression of the *SOX2* gene in mesenchymal stem cells of Wharton’s jelly at the transcript level. The study also examined the effect of a patient’s age, pregnancy length, birth weight and cord blood parameters on SOX2 gene expression in the examined material.

## 2. Results

### 2.1. Cell Culture and Cytometric Analysis

**Figure 1:**
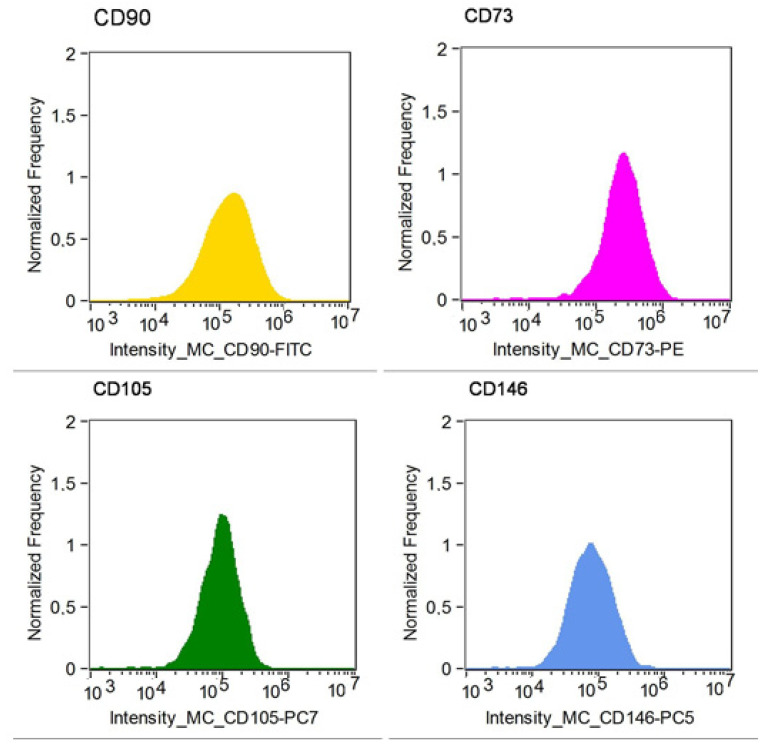
Expression of CD73, CD90, CD105, and CD146 surface antigens in Wharton’s jelly stem cells. Cytometric analysis carried out on a MoFlo XDP cell sorter (Beckman Coulter). Analysis of minimum 10,000 events was recorded for each probe.

**Figure 2:**
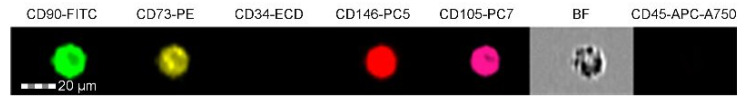
Single stem cells, antigens (CD90, CD73, CD146, CD105 positive and CD34, CD45-negative). Photographs of single samples of cells, presenting microscope image (BF) and fluorescence in channels, showing the expression of studied antigens (FlowSight f. Amnis flow cytometer).

**Figure 3:**
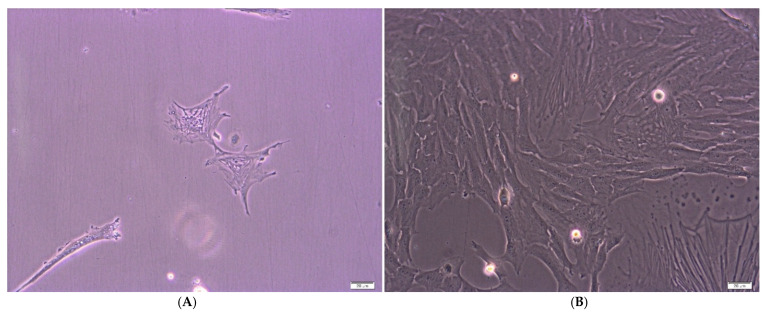
(**A**). Stem cells from a 4-day culture (**B**). Stem cells from a 10-day culture; bright field microscopy (BF), 100× magnification (Xcellence RT system with an IX81 inverted microscope Olympus).

Using flow cytometry (Figure 1 and Figure 2) and cell culture under adhering conditions (Figure 3A,B), mesenchymal character of the analyzed cells was demonstrated. The cytometric test confirmed the presence of surface antigens characteristic for MSC on the tested cells, such as: CD73, CD90, CD105 and CD145. During cell culture, the adherence capacity to the plastic walls and the fibroblast-like shape of the analyzed cells were confirmed.

### 2.2. SOX2 Gene Expression Analysis

**Figure 4:**
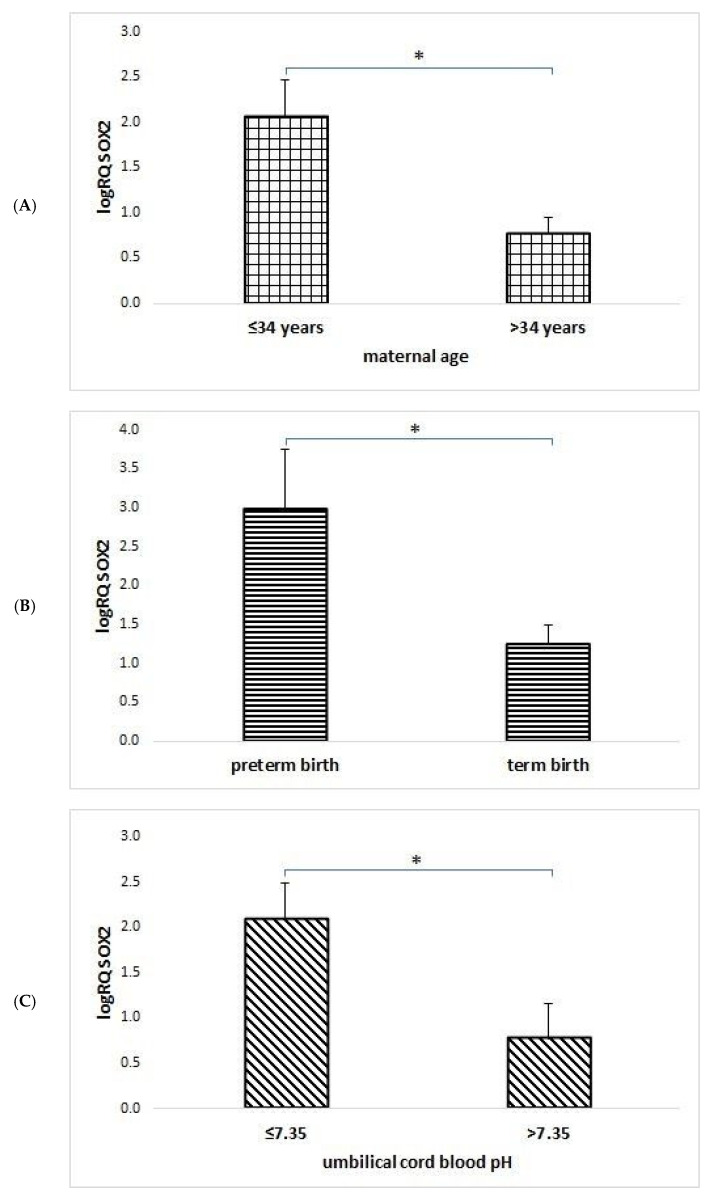
Average expression of *SOX2* gene (logRQ ± SE) in WJSC (**A**). depending on the parturient’s age; (**B**). depending on delivery time; (**C**). depending on umbilical cord blood pH. *, *p* < 0.05 *U* Mann Whitney test.

**Figure 5:**
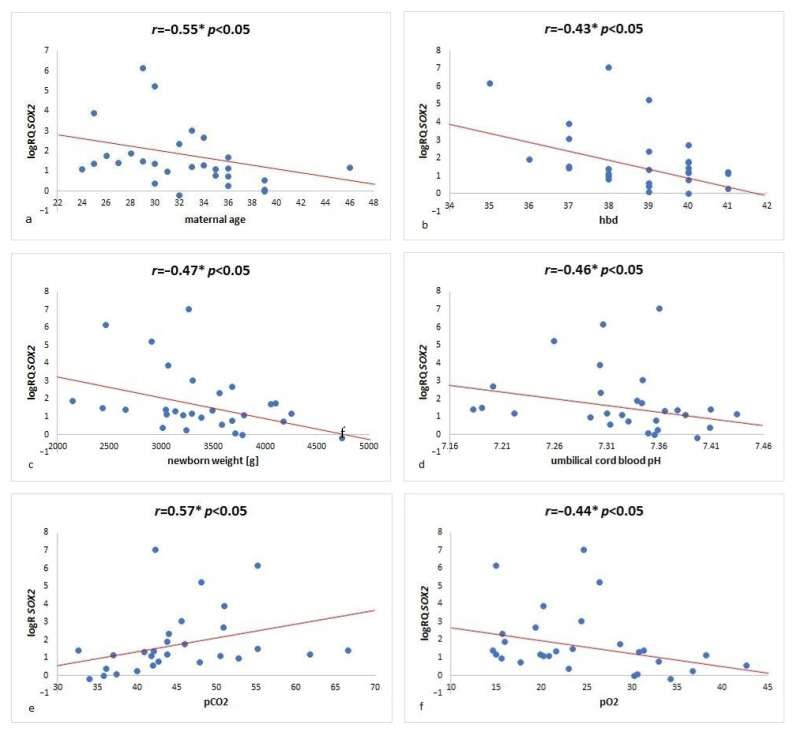
*SOX2* gene expression (logRQ) on (**a**) the parturient’s age (**b**) the week of pregnancy in which the birth took place, (**c**) newborn’s birth weight, (**d**) pH of the umbilical cord blood, (**e**) carbon dioxide pressure, (**f**) oxygen pressure. * The Spearman Rank Order.

The presence of the *SOX2* gene transcript was shown in all examined mesenchymal Wharton’s jelly stem cells. The results showed that the expression of the *SOX2* gene in WJSC varies significantly depending on maternal age. In women aged 34 years and younger, significantly higher expression of the *SOX2* gene was recorded in comparison to women over 34 years of age (*p* = 0.005) (Figure 4A). The analysis of the correlation between gestational age and the *SOX2* gene expression revealed a negative correlation (r = −0.55, *p* < 0.05) (Figure 5a). Significant differences in the expression of the *SOX2* gene in WJSC have been noted in relation to the time of delivery. In the group of women who gave birth before the due date, the expression of the *SOX2* gene in Wharton’s jelly mesenchymal stem cells was statistically significantly higher compared to women who gave birth in due course (*p* = 0.002) (Figure 4B). An analysis of a correlation between the *SOX2* gene expression and the week of pregnancy in which the birth took place discovered a significant negative correlation (r = −0.43, *p* < 0.05) (Figure 5b). A statistically significant negative correlation between the *SOX2* gene expression level and the birth weight (r = −0.47, *p* < 0.05) was noted (Figure 5c). An analysis of the *SOX2* gene expression in relation to the umbilical cord blood pH showed a significantly negative correlation between the *SOX2* gene expression level and the cord blood pH (r = −0.46, *p* < 0.05) (Figure 5d). Furthermore, it was observed that the *SOX2* expression in WJSC is statistically significantly higher at pH 7.35 (*p* = 0.02) or lower (Figure 4c). An analysis of the *SOX2* gene expression in relation to the oxygen and carbon dioxide pressure showed a statistically significant negative correlation of the *SOX2* gene expression with pO_2_ (r = −0.44, *p* < 0.05) (Figure 5f) and a statistically significant positive correlation of the *SOX2* expression level with pCO_2_ (r = 0.57, *p* < 0.05) (Figure 5e). The investigation did not show the impact of the delivery route, drugs used during pregnancy and delivery on the *SOX2* gene expression in WJSC.

## 3. Discussion

The authors present the study showing the expression of the *SOX2* gene, regarded as one of the main factors of Wharton’s jelly stem cell pluripotency factors to be contingent on the parturient’s age, the maternal age, birth weight, the pH of the umbilical cord blood, carbon dioxide pressure and oxygen pressure. 

The *SOX2* expression in WJSC was statistically significantly higher at the pH of umbilical cord blood equal to or lower than 7.35. They also have shown a correlation between the *SOX2* gene expression and the physicochemical parameters of umbilical cord blood. The *SOX 2* expression was increased at lower O_2_ and higher CO_2_ levels of umbilical cord blood. The *SOX2* level was increased with a decrease in cord blood pH. Obradovic et al. showed that Wharton’s jelly stem cells cultured in vitro at 3% oxygen concentration showed higher expression of *SOX2* compared to cells cultured at 21% oxygen concentration. It was also noted that lower oxygen concentration increases in vitro migration ability of culture and enhances the activity of proteolytic enzymes, as well as protecting the cell from harmful factors. The authors theorize that low oxygen concentration enhances WJ-MSC multipotency by stimulating their self-renewal and increasing the expression of the pluripotency factor which can boost the therapeutic potential of WJSC [46]. As previously explained in the literature, it has been shown that higher SOX2 expression in stem cells is associated with stemness, greater self-reinforcement potential, better proliferative properties, and probably also increases cell migration and adhesion, and influences their paracrine properties [28,29,30,31].

Mesenchymal stem cells require adequate oxygen concentration for their physiological function. The balance between differentiation, apoptosis and self-mood which is characteristic of stem cells must be achieved through regulation by the microenvironmental niche in which stem cells reside. Oxygen concentration is an important factor to consider when growing stem cells in tissue engineering and regenerative medicine [47]. In their research, Halim et al. focused on finding a combination of factors in vitro necessary to control stem cell proliferation, which would allow them to remain viable and undifferentiated, by analyzing, among other things, oxygen concentration [48]. The research conducted by Widowati et al. showed that Wharton’s jelly MSCs, cultured under hypoxic conditions, have a higher rate of proliferation but show no difference in surface markers from cells grown under normoxic conditions [49]. Yamamoto et al. demonstrated that low oxygen pressure enhances proliferation and increases the number of growth factors secreted by stem cells derived from adipose tissue. The authors would like to emphasize the effectiveness of hypoxic cultures for ASC expansion and maintenance of an undifferentiated state for further therapeutic use [50]. Zhao et al. show that HSPCs of the umbilical cord blood maintain stemness better under hypoxic conditions [51]. It is difficult to compare our research with the research presented in the literature, due to the use of various units by the authors, moreover, in our study, we assess the possible effect of the pressure of carbon dioxide and oxygen in the umbilical cord blood on the level of *SOX2* expression in MSC, while the level of CO_2_ and O_2_ in culture cellularity was constant at 5% and 15%. Safitri et al. studied the effect of oxygen concentration on the level of SOX2 expression in the MSC of rabbit bone marrow. They observed that low in vitro oxygen pressure conditions increased *OCT4* and *SOX2* expression compared to conventional or hyperoxic conditions [52]. Bae et al. suggest that *SOX2* is a gene that is exceptionally amplified under hypoxic conditions [53]. The presented research agrees with findings from other studies. It demonstrates that lower oxygen pressure is linked to higher *SOX2* gene expression. Our results indicate that mesenchymal stem cells show higher expression of the *SOX2* gene in a more acidic environment with lower oxygen pressure and higher carbon dioxide pressure. We imply that in vivo the pH of umbilical cord blood, oxygen and carbon dioxide concentration are important factors regulating stem cells by influencing *SOX2* expression. This fact may prove to be valuable information used in the stem cell collection process as well as during the handling of the cells. 

The authors have demonstrated that the expression of the *SOX2* gene in WJSC is statistically significantly higher in women aged 34 years and younger compared to women over 34 years of age. It is also shown that there is a statistically significant moderate negative correlation between maternal age and the *SOX2* gene expression. It was established in several studies that the parturient’s age affects the quantity and quality of stem cells, however, the studies focused mainly on cord blood stem cells [36,39,40,54]. Alrefaei et al. evaluated the effect of maternal age on the expression of the mesenchymal stem cell markers CD105 and CD29 in various regions of the human umbilical cord and showed that there were significant negative correlations between maternal age and CD29 and CD105 expression [55]. In studies with rats, Asmuda et al. observed that the expression profile of Sox-2 in bone marrow derived MSCs of old rats was significantly lower compared to that of young rats [56]. Huang et al. suggested that the younger donor umbilical cord is a relatively effective source of MSC. The authors speculated that the older donor’s umbilical cord cells showed reduced differentiation capacity, and this could be attributed to the decreased functional status of the older maternal organs, which play a supporting role and the microenvironment enabling the development of umbilical cord MSC [57].

No studies have been found with regard to the influence of the parturient’s age on the *SOX2* expression level in WJSC. In the authors’ previous research, it was shown that the expression of the *POU5F1* gene [58] and the expression of the *BIRC2*, *BIRC3* and *BIRC5* genes [59] decrease with maternal age. It was noted that the expression of the *SOX2* gene is statistically significantly higher in WJSC of babies born prematurely, and the level of the *SOX2* expression correlated positively with the length of pregnancy. The earlier the birth took place the higher the *SOX2* expression in WJSC. The study also showed a negative correlation between the *SOX2* expression and the birth weight. It was observed that the lower the birth weight, the higher the *SOX2* gene expression. Researching the influence of the birth weight and the time of delivery on the quality of stem cells is focused mainly on the umbilical cord blood. A number of studies have demonstrated that higher birth weight and, consequently, a larger volume of the placenta, acts as a stimulus on the number of stem cells in the umbilical cord blood [36,38,39,40,60]. On the other hand, other researchers note that the size of the placenta is related to the number of pregnancies, and so the first pregnancy is usually associated with the weakening of the vascularization of the placenta, while the more births, the larger the size of the placenta in multi-family mothers, thus providing a greater volume of umbilical cord blood and more cells. CD34 [61,62]. However, these studies looked only at the number of stem cell

Looking at the delivery time, the reports vary. Some studies report a high number of stem cells collected during term deliveries [34]. Others suggest that during preterm births the number of CD34+ cord blood cells is higher compared to the predicted due date [33,63]. In our study, we accepted preterm deliveries before our 37th week of pregnancy. The earliest born child was at 35 weeks of pregnancy. However, no studies on the influence of birth weight and delivery time on WJSC quality or expression of the *SOX2* gene in WJSC have been found in the available literature.

Low birth weight may result from preterm birth and/or intrauterine growth restriction (IUGR). Premature birth (PT) and low birth weight (LBW) are associated with numerous health and social consequences, both short-term and long-term. These infants have an increased risk of death in the perinatal period and are at increased risk of developing chronic diseases [64]. According to some authors these two adverse pregnancy outcomes, PT and LBW should be investigated together [65]. Kotowski et al. found that the count of cord blood non-HSCs/VSELs is inversely associated with the birth weight of preterm infants. They also noticed that a high number of cord blood HSCs is strongly associated with a lower risk of prematurity complications [66]. The conducted research provides valuable information in the context of possible compensation mechanisms for babies born prematurely and with low birth weight. Further research is needed to evaluate the umbilical cord in babies with low birth weight and premature births, due to the possible better therapeutic potential of the collected cells with regard to increased expression of the *SOX2* gene.

In the author’s previous work, it was demonstrated that the expression of the second key factor responsible for maintaining the state of pluripotency, *POU5F1* in stem cells of Wharton’s jelly cord, is dependent on the age of the gravida, the manner of delivery, the method of delivery and the use of oxytocin. MSCs derived from Wharton’s jelly (WJSC) taken from younger women and during their first childbirth as well as from patients who received oxytocin showed higher expression of *POU5F1* [58]. In the authors’ previous studies, it was also observed that MSCs derived from Wharton’s jelly (WJSC) collected from younger women who were giving birth naturally and in an acidic umbilical cord blood environment are characterized by higher expression of inhibitor of apoptosis protein (IAP): *BIRC2, BIRC3* and *BIRC5* genes, making them more resistant to apoptosis. IAPs have multidirectional effects and a wide range of cellular functions; in addition to promoting cell survival, they are also involved in signal transduction, cell differentiation, cell response to damage, and cell division [59].

## 4. Conclusions

The authors concluded that the younger the woman and the earlier the birth takes place, the lower the birth weight and the higher the *SOX2* gene expression in WJSC. In addition, it has been noticed that the physicochemical parameters of umbilical cord blood, such as O_2_, pressure, CO_2_ pressure and pH are the factors that regulate the expression of the *SOX2* gene in WJSC. However, the correlation coefficients obtained in our study, although significant, are quite low (around 0.5), thus, the study should be continued with a larger number of patients. Due to functions performed by the *SOX2* in stem cell biology, it is possible to draw a conclusion that increased expression is likely to translate to higher stem cell effectiveness. Stem cells with high *SOX2* expression have a lower degree of differentiation. They have higher proliferative potential, which makes them more clinically useful. Our findings look promising and warrant the need for further research.

## 5. Materials and Methods

The study was conducted on a group of 30 women hospitalized in the Department of Obstetrics and Pathology of Pregnancy of the Independent Public Clinical Hospital No. 1 in Lublin. The age range of the patients was 24–46 years. The women had a section of the umbilical cord sampled soon after delivery. The statistics with regard to the examined group are presented in Table 1. The research was carried out with the consent of the Bioethics Committee at the Medical University of Lublin no. KE-0254/128/2014. All methods were carried out in accordance with relevant guidelines and regulations. Informed consent was obtained from all subjects and/or their legal guardian(s).

The study was carried out on mesenchymal stem cells of umbilical cord Wharton’s jelly. The stem cells were obtained using the explant method. A section of an umbilical cord removed soon after delivery was placed in a sterile container with an antibiotic and culture medium. Next, the umbilical cord was sectioned into smaller fragments in the laboratory and cultured for 10 days. Cell culture conditions: culture medium: DMEM (1×) + GlutaMAX[+] 1 g/L D-Glucos [+], Pyruvate; Gibco, Paisley, UK; Serum: Heat Inactivated FBS; Gibco, Carlsbad, CA, USA; Antibiotics: Amphotericin B 250 μg/mL + Penicillin/Streptomycin (100×); PAA, Austria; Temperature: 37 °C; O_2_ concentration: 15%; CO_2_ concentration: 5%. The isolation procedure is described in the authors’ previous work [67]. The obtained cells were subjected to a cytometric analysis in order to confirm mesenchymal character using antibodies against CD73 (PE-Cy7-labeled), CD90 (FTC-labeled) and CD105 (C7-labeled), CD146 (C5-labeled), CD34 (ECD), and CD45 (APC-A750-labeled) surface antigens (DURAClone SC Mesenchymal Tube, BeckmanCoulter, France). The procedure of cytometric analysis is presented in paper [68]. Total cellular RNA was isolated from the cells using the modified method of Chomczyński and Sacchi [69]. The following reagents were used for the isolation: TRI Reagent (Sigma-Aldrich, St Louis, MO, USA), Chloroform (Sigma-Aldrich, USA), Isopropanol (Sigma-Aldrich, USA) and Ethanol (Poch, Poland). Subsequently, reverse transcription was performed to obtain complementary DNA. The cDNA synthesis was carried out in accordance with the manufacturer’s recommendation using the High-Capacity cDNA Transcription Kits (Applied Biosystems, Foster City, CA, USA). The synthesized cDNA was amplified in the qPCR reaction, using commercially available TaqMan Hs0153049_s1 probes for the SOX2 gene and Hs99999905_m1 for GAPDH endogenous control (Applied Biosystems, Foster City, CA, USA). The analysis of the obtained results was performed using Expression Suite Software. The ΔΔCt method [70] was used to determine the relative level of expression of the examined gene. The statistical analysis was performed using Statistica 13 software. Mann Whitney’s U test was used to assess the differences in the studied groups. The correlation was assessed with Spearman’s rank test. The *p*-value was set at *p* < 0.05.

## Figures and Tables

**Table 1:** Statistics of pregnancy related factors.

Parameter	N	Mean	Median	Minimum	Maximum	SD
maternal age [years]	30	32.3	32.000	24.000	46.000	5.098
number of pregnancies		2.000	2.000	1.000	8.000	1.414
week of pregnancy		38.786	39.000	35.000	41.000	1.548
number of deliveries		1.862	2.000	1.000	7.000	1.187
newborn’s weight [g]		3383.793	3300.000	2140.000	4740.000	584.089
pH *		7.326	7.341	7.182	7.434	0.066
pCO_2_ [mmHg] *		45.268	43.800	32.600	66.500	8.098
PO_2_ [mmHg] *		24.643	23.200	14.600	42.700	7.887
cHCO_3_ [mmol/L] *		22.729	23.100	19.200	26.700	2.045

* Blood acid-base balance indicators were determined on an ABL90 FLEX gas analyzer (Radiometer, Denmark).

## Data Availability

The datasets used and/or analyzed during the current study are available from the corresponding author on reasonable request.
